# Biomimetic PLGA/Strontium-Zinc Nano Hydroxyapatite Composite Scaffolds for Bone Regeneration

**DOI:** 10.3390/jfb13010013

**Published:** 2022-01-28

**Authors:** Mozan Hassan, Mohsin Sulaiman, Priya Dharshini Yuvaraju, Emmanuel Galiwango, Ihtesham ur Rehman, Ali H. Al-Marzouqi, Abbas Khaleel, Sahar Mohsin

**Affiliations:** 1 Department of Anatomy, College of Medicine and Health Sciences, United Arab Emirates University, Al Ain P.O. Box 15551, United Arab Emirates; 202090098@uaeu.ac.ae (M.H.); mohsins.sulaiman@uaeu.ac.ae (M.S.); 2 Department of Pharmacology, College of Medicine and Health Sciences, United Arab Emirates University, Al Ain P.O. Box 15551, United Arab Emirates; priyay@uaeu.ac.ae; 3 Department of Chemical and Petroleum Engineering, College of Engineering, United Arab Emirates University, Al Ain P.O. Box 15551, United Arab Emirates; emmanuel.galiwango@ontariotechu.ca (E.G.); hassana@uaeu.ac.ae (A.H.A.-M.); 4 Energy Systems and Nuclear Science Faculty, Ontario Tech University, Oshawa, ON L1G 8C4, Canada; 5 Engineering Department, Faculty of Science and Technology, Lancaster University, Gillow Avenue, Lancaster LA1 4YW, UK; i.u.rehman@lancaster.ac.uk; 6 Department of Chemistry, College of Science, United Arab Emirates University, Al Ain P.O. Box 15551, United Arab Emirates; abbask@uaeu.ac.ae

**Keywords:** PLGA, bone scaffolds, zinc, strontium, nano-hydroxyapatite, supercritical CO_2_

## Abstract

Synthetic bone graft substitutes have attracted increasing attention in tissue engineering. This study aimed to fabricate a novel, bioactive, porous scaffold that can be used as a bone substitute. Strontium and zinc doped nano-hydroxyapatite (Sr/Zn n-HAp) were synthesized by a water-based sol-gel technique. Sr/Zn n-HAp and poly (lactide-co-glycolide) (PLGA) were used to fabricate composite scaffolds by supercritical carbon dioxide technique. FTIR, XRD, TEM, SEM, and TGA were used to characterize Sr/Zn n-HAp and the composite scaffolds. The synthesized scaffolds were adequately porous with an average pore size range between 189 to 406 µm. The scaffolds demonstrated bioactive behavior by forming crystals when immersed in the simulated body fluid. The scaffolds after immersing in Tris/HCl buffer increased the pH value of the medium, establishing their favorable biodegradable behavior. ICP-MS study for the scaffolds detected the presence of Sr, Ca, and Zn ions in the SBF within the first week, which would augment osseointegration if implanted in the body. nHAp and their composites (PLGA-nHAp) showed ultimate compressive strength ranging between 0.4–19.8 MPa. A 2.5% Sr/Zn substituted nHAp-PLGA composite showed a compressive behavior resembling that of cancellous bone indicating it as a good candidate for cancellous bone substitute.

## 1. Introduction

Bone tissue engineering (BTE) is a promising strategy that can be used to regenerate and replace injured bones and to compensate the conventional clinically used synthetic bone grafts that have many limitations such as immune rejection, infection, and prolonged hospitalization [1]. BTE involves adopting various biomaterials to act as a bone scaffold [2], especially in critical-size bone defects of 2.5 cm or greater that would not heal naturally and require surgical intervention [3,4]. A tremendous amount of work in BTE has been reported in the last decade, but still, scaffolds manufactured cost-effectively, mimicking natural bone tissue, promoting osteoinductivity, and, in addition, sufficiently porous to provide adequate vascularization for oxygen, nutrient supply, and waste excretion are still a challenge [5]. Simultaneously, scaffolds should provide adequate mechanical strength and temporary support to the cells, such as those of native bone tissue, and at the same time possess an appropriate degradation rate tailored to tissue growth and bone formation [6,7]. Scaffolds used in bone tissue engineering can be from natural or synthetic polymers or bioceramics. Natural polymers such as collagen [8], silk [9], chitosan [10], and alginate [11] have been used in bone tissue engineering. These are known to cause a minimal immune reaction, but at the same time, their use is hindered due to reduced mechanical stability [12]. Synthetic polymers such as polycaprolactone (PCL), poly-lactic acid (PLA), and poly lactic-co-glycolic acid (PLGA) are biocompatible and have controllable degradation rates [12].

PLGA is a linear copolymer and FDA-approved material used preferably in bone tissue engineering. It is characterized by good mechanical properties and is composed of different ratios of lactic acid and glycolic acid; these ratios can be manipulated to adjust the scaffold degradation rate. However, the use of pure PLGA polymer has been limited due to its reduced osteoinductivity; as a result, PLGA is usually used in combination with composite materials such as bioceramics [2]. Hydroxyapatite (Ca_10_(PO_4_)_6_(OH)_2_ (HA)) materials have been introduced as bone substitutes due to their ability to enhance osteoinductivity and their bioactivity, which results from their structural similarity to natural bones [13]. Natural bones are composed of 70% inorganic phase comprising mainly HA with mineral trace elements such as iron, copper, zinc, manganese, fluoride, strontium, and boron, and 30% organic material, mainly collagen fibers assembled within the HA matrix [14]. One of the major drawbacks of HA, when used as a single phase in the fabrication of scaffolds, is its low stiffness and mechanical strength and poor osteoconductivity [15]; therefore, metal ion substitution has been used to enhance the scaffold biological activity and performance [16].

Enhancing hydroxyapatite with magnetic nanoparticles is widely used in various biomedical applications [17]. Magnetic scaffolds impregnated with iron oxide are used for bone regeneration but need a magnetic field for stimulation of the magnetic particles, and it is reported that the magnetic nanoparticles may not disperse well and penetrate inside the three-dimensional scaffold, decreasing the magnetic field stimulus, so the magnetic particles tend to agglomerate with time, and the application of the external magnetic field need to be adjusted, as it can affect the cellular morphology [18,19]. 

Biological hydroxyapatite contains trace amounts of metal ions such as Mg, Mn, Zn, and Sr that are important for bone biological and metabolic activities [20]. Several studies investigated the substitution of Ca, P, or OH groups of hydroxyapatite with these metal ions to improve scaffold biological response and physical properties [21]. HA structural stability permits the substitution of ions such as strontium (Sr), manganese (Mn), and zinc (Zn) to replace Ca or (PO_4_)^3−^ [22]. These metal ions play an important role in various signaling and metabolic pathways and act as a cofactor for several enzymes; additionally, they are known to enhance osteogenic gene differentiation [23]. Among these ions, Zn is involved in many biological functions, and its incorporation into HA was reported to enhance bone formation and accelerate wound healing due to its anti-inflammatory properties. It is reported that Zn deficiency can reduce bone density and make bone susceptible to fractures [24]. Sr was also reported to enhance bone formation by increasing osteoblastic activity and at the same time decreasing bone resorption [25]. Furthermore, Aoki et al. showed that Sr-doped HAp has better mechanical properties compared to HAp [26]. Qamar et al. also showed that-Zn doped HAp enhanced scaffold mechanical properties without altering scaffold bioactivity [27]. The present study sought to fabricate and characterize a multifunctional hybrid scaffold with adequate mechanical properties to be used as a bone scaffold. To overcome the drawbacks of using single-phase HA and PLGA in a scaffold and enhance the biological and mechanical properties, a composite scaffold of different ratios of Sr/Zn substituted HA and PLGA was fabricated in this study using a simple supercritical carbon dioxide technique (scCO_2_). The ScCO_2_ technique is an organic non-solvating process in which porous structures of polymers can be achieved under low temperatures. ScCO_2_ is nonflammable, non-toxic, inexpensive, and soluble in polymers [28,29]. To optimize the degree of zinc and strontium substitution in n-HAP at a rate so as not to mitigate strength was another main objective of the study, as to our knowledge, only a limited number of studies have reported on Sr- and Zn-doped n-HAp incorporated PLGA scaffolds for bone tissue regeneration. The synthesized scaffolds fulfilled the well-defined ideal criteria for scaffolds as they were bioactive, biodegradable, mechanically stable, and sufficiently porous. They are capable of releasing strontium and zinc ions once implanted in vivo, which will facilitate early osseointegration.

## 2. Materials and Methods

### 2.1. Synthesis of Nano Hydroxyapatite 

Calcium nitrate tetrahydrate Ca(NO_3_)_2_∙4H_2_O, 98%, Merck PROLABO (Darmstadt, Germany) (0.4 M) and ammonium dibasic phosphate (NH_4_)_2_HPO_4_, 99%, Merck Chemicals Co., (Darmstadt, Germany) (0.2 M) were dissolved in de-ionized water separately [30]. An ammonium dibasic phosphate solution was added dropwise to the calcium nitrate solution under vigorous stirring (750 rpm) to achieve precipitation. The pH of the reaction mixture was adjusted to pH 11 by adding concentrated sodium hydroxide (NaOH, 99%, Merck Chemicals Co.). The formed calcium phosphate precipitate (molar ratio of Ca/P of 1.67) was aged overnight at room temperature, centrifuged, and washed thrice with de-ionized water. The resulting powder was dried in an air oven at 100 °C and calcinated at 1000 °C in the air for 1 h with a heating rate of 5 °C/min and then cooled at the same rate in the air.

### 2.2. Preparation and Characterization of n-HAp, Sr-n-HAp, Zn-n-HAp and Sr-Zn-n-HAp 

Aqueous solutions of the synthesized n-HAp (1%) were mixed with strontium nitrate (Sr(NO_3_)_2_, ≥99.0%, Merck Chemicals Co) and zinc nitrate hexahydrate (Zn(NO_3_)_2_∙6H_2_O, Merck Chemicals Co) (0.001, 0.0025, and 0.004 M) to prepare 1, 2.5, and 4 mol% Sr-doped n-HAp and Zn-doped n-HAp, respectively (Table 1). The (Ca+Sr)/P and (Ca+Zn)/P molar ratios were kept constant at 1.67 throughout the experiments [31,32]. The mixed solutions were stirred for 1 h at room temperature. The obtained suspensions were filtered and dried in a hot air oven at 80 °C for 72 h.

The Master particle size analyzer (Mastersizer E) was used to measure the particle size of the prepared powders; particle size distributions are presented as D-values (d10, d50, and d90) [33]. 

The structure of the prepared material, including phase composition and crystallinity, was analyzed using an X-ray diffractometer (Shimadzu Lab X XRD-6100 Diffractometer, Kyoto, Japan). The XRD data were obtained at a 2θ angle ranging from 20° to 80° at a scanning speed of 0.02°/min, 40 kV, and 30 mA, and obtained patterns were then compared with standards established by the Joint Committee on Powder Diffraction and Standards (JCDPS) [34].

Fourier transform infrared spectroscopy (FTIR) (Thermo Fischer Nicolet Nexus 470 FT-IR 0 (Waltham, MA, USA) was used to determine the structure and chemical bonds of the powders. Spectra were recorded with a resolution at 4 cm^−1^ over a scan range of 400 cm^−1^ to 4000 cm^−1^ with an average of 50 scans. 

The powders were dispersed well in ethanol by a sonication process for 15 min, before being dropped onto the carbon-coated copper grids. They were air-dried to characterize the morphology using transmission electron microscope (FEI Spirit G2 BioTwin, Eindhoven, The Netherlands, Tecnai 200 KV), HT = 100 kV, spot size = 1 magnification = 50 nm. Thin foil apertures: condenser aperture = 100 µm; objective aperture = 40 µm).

### 2.3. Fabrication of Sr/Zn-n-HAp Incorporated PLGA Composite Scaffolds

Composite scaffolds consisting of PLGA-incorporated Sr/Zn-n-HAp were fabricated by supercritical CO_2_ technique [35]. Different ratios of PLGA, Sr, Zn, and n-Hap were used to fabricate four types of bone scaffolds (Table 2).

PLGA (lactide:glycolide, 75:25, mol wt 66,000–107,000, Sigma Aldrich, Saint Louis, MO, USA was dissolved in 10 mL of hexafluoro propanol (HFP), and mixed on a magnetic stirrer at room temperature for 15 min. Thereafter, 1 mL of Sr/Zn-nHAp dispersions in HPF were added dropwise into PLGA/HFP solution under stirring at 1200 rpm for 30 min, followed by precipitation with the addition of 15 mL ethanol [36]. The solvent was evaporated, and the PLGA- Sr/Zn-nHAp composite was formed by freeze-drying the prepared polymer solutions for 2 days.

An extraction system ISCO SFX-220 (Lincoln, NE, USA) consisting of an SFX-220 extractor, an SFX-200 controller, and a carbon dioxide cylinder with a siphon output was used to generate porous composite powder. The composite (200 mg) was added into a custom-made cylindrical mold (Supplementary Figure S1). The mold was placed into a thread-sealed high-pressure vessel with a volume of 80 mL, equipped with a super thermostat (CH-1015, Hengping Instrument and Meter Factory, Shanghai, China) and a custom-made pressure transducer for the control of temperature and pressure, respectively. A temperature of 35 °C and pressure of 250 bar were maintained for over 20 min in a vessel. High-pressure CO_2_ was flushed into the vessel (10 mL/min) until the saturation pressure was reached, and the pressure and temperature in the vessel were maintained constant for 60 min. Then, the pressure within the vessel was decreased rapidly to ambient pressure, and the porous scaffold was obtained [37].

### 2.4. Characterization of Composite Scaffolds

The scaffolds were characterized by X-ray diffraction (XRD), Fourier-transform infrared spectroscopy (FTIR), and thermogravimetric analysis (TGA). To understand the structural changes upon heating of the composite scaffolds, TGA was carried out at 50–600 °C in the air at a heating rate of 10 °C/min and flow rate of 40 mL/min using a thermogravimetric analyzer (TGA-50, Shimadzu, Koyoto, Japan).

The bioactivity of the scaffolds was determined by the formation of an apatite-like phase in simulated body fluid (SBF). Reagents for preparation of simulated body fluid (SBF) solution [29]: sodium chloride (NaCl), sodium hydrogen carbonate (NaHCO_3_), potassium chloride (KCL), di-potassium hydrogen phosphate trihydrate (K_2_HPO_4_∙3H_2_O), magnesium chloride hexahydrate (MgCL_2_∙6H_2_O), calcium chloride (CaCl_2_), sodium sulphate (Na_2_SO_4_), tris-hydroxymethyl aminomethane (TRIS) ((HOCH_2_)_3_CNH_2_), 1 M HCL solution. A solution of SBF was prepared according to the published method [38]. The composite scaffold samples were weighed, and the amount of SBF required for each sample was calculated according to the equation: Volume of SBF needed = sample weight (g) × 50 mL/0.075 g.

Flasks containing the samples were placed in an agitator at 37 °C, and the pH value was adjusted to 7.4. The solution was filtered at 8 h, 24 h, 120 h, and 336 h. The filtered scaffold material was sterilized by washing with ethanol. SEM was performed on these samples to assess the bioactivity of the scaffolds by the formation of apatite crystals [38].

The effect of Sr/Zn-nHAp addition on the degradation behavior of PLGA was determined by their change of pH values and percentage weight loss in SBF. The samples were incubated at 37 °C for 2 weeks. The scaffolds were removed from SBF at different time points (5, 10, 15, 20, 25, 30, 35, 40, 45 days), and dried at 50 °C. The percentage of weight loss was determined using the following formula:

Weight loss = W_0_ − W*_t_*/ W_0_ × 100

where W_0_ was the initial weight and W*_t_* represented the dry weight at time *t*.

The pH of the sample was measured with a pH meter (Thermo scientific, Orion Star, A211, Waltham, MA, USA). The releases of Ca^2+^, Sr^2+^, and Zn^2+^ from the collected filtered SBF solution were quantified at different time points using inductively coupled plasma mass spectrometry (ICP-MS; NexION 300X, from PerkinElmer, Massachusetts, USA). ICP-MS analysis was performed by preparing calibration standards using an SBF solution to have a comparable ionic strength of calibration standards and sample solutions. The samples were prepared by diluting (1:100000) with 2% nitric acid prepared in deionized water to prevent precipitation throughout the ICP-MS analysis.

Scanning electron microscopy (SEM; JSM-6010PLUS, JEOL, Tokyo, Japan) was conducted on 5 mm size scaffold samples mounted on an aluminum stub platform and coated with gold. A minimum of six areas were scanned for each sample. The obtained images were examined by ImageJ software to determine the distribution of pores and to calculate the average pore size [39].

### 2.5. Mechanical Testing of the Scaffolds

Different scaffolds (nHAp, PLGA-nHAp, PLGA-1%Zn/Sr-nHAp, PLGA-2.5% Zn/Sr-nHAp, and PLGA-4% Zn/Sr-nHAp) of cylindrical shape were prepared using an iron mold of dimension 64.5 mm height and 36 mm diameter. The composite powder was placed in the mold and pressed in a Carver Bench Top Press (Carver Inc., Wabash, IN, USA, Model 4386, S/N 170357) under room temperature with 22000 lb. pressure for 15 min, then the pressure was released to remove the scaffold from the mold. Samples were prepared with an aspect ratio of 1 and subjected to mechanical testing (see Supplementary Figure S1). 

Samples were mechanically tested under compression with a displacement velocity of 2mm/min using the Universal Testing System Instron 5960 equipped with a 5KN load cell. Scaffolds were placed in the center of the loading frame to ensure uniform loading. A stress-strain response was derived from the force-displacement curve by dividing the force by the cross-sectional surface area and the displacement by the initial length of the sample. The ultimate strength was calculated from the peak of the curve, after which the line started to decline, i.e., the scaffold started to collapse.

### 2.6. Statistical Analysis

Data for the pore sizes were presented as mean ± standard error of the mean (SEM). One-way analysis of variance (ANOVA) with Bonferroni’s multiple comparison test was carried out in GraphPad Prism 6.0. The ultimate strength was expressed as mean ± standard error of the mean (SEM). A *t*-test was used to determine the significant differences between the means. *p* < 0.05 was considered to be statistically significant. 

## 3. Results and Discussion

### 3.1. Characterization of nHAp, Sr-nHAp, Zn-nHAp, Sr/Zn-nHAp

#### 3.1.1. Particle Size Analysis

The average particle size (d50) of pure HAp powder was 92.3 nm, while more than 90% of particles (d90) were 196.4 nm. The substitution of 1% of Sr and Zn to nHAp resulted in a slight reduction of average particle size from 92.3 to 86.0 nm (Table 3 and Supplementary Figure S2). The average particle size remained at 86.0 nm when the strontium and zinc concentration was increased to 2.5%. However, Sr and Zn substitution up to 4% in the nHAp decreased the average particle size, hence causing structural changes within the lattice structure of the HA. A reduction of both the particle size and degree of crystallinity after Ca substitution by Sr, Zn, and Mg was also reported in earlier studies [24,40]. Bioceramics with nanostructured surfaces and strontium substituted hydroxyapatite composites promote adsorption of protein, proliferation of osteoblasts, differentiation of osteocytes, and bone formation in vivo [41]. 

#### 3.1.2. X-ray Diffraction Analysis 

**Figure 1:**
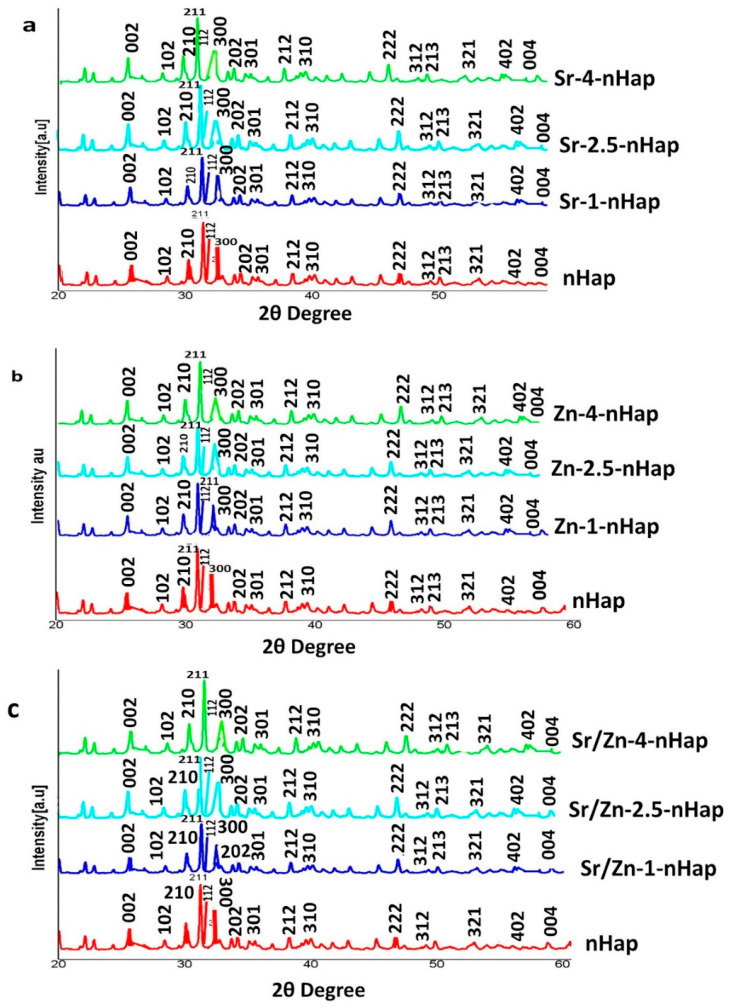
XRD pattern of Sr-nHAp (**a**), Zn-nHAp (**b**), Sr/Zn-nHAp (**c**).

The X-ray diffraction (XRD) patterns of the powders are shown in Figure 1. XRD analysis showed characteristic diffraction patterns of hydroxyapatite. The pattern presented broad peaks in the planes (002), (210), (211), (112), (300), (202), (310), and (222), which indicated that nHAp crystallizes well [42]. These peaks were indexed according to the standard pattern (JCPDS 09-0432). The peaks of Sr-nHAp and Zn-nHAp also exhibited significant broadening. Compared to the XRD pattern of pure nHAp, the peak positions of Sr-doped nHAp were shifted slightly to lower 2θ values and showed broadening (Figure 1a). XRD peaks for metal-doped hydroxyapatite showed broadened peaks with a shift to lower 2θ values, indicating decreased crystallinity of the samples and effective replacement of calcium by larger ions within the nHAp lattice [43]. A broadened peak with a small switch to greater 2θ values was observed for the Zn-4-nHAp, as shown in Figure 1b. The peak shift to the right in a high concentration of zinc-doped hydroxyapatite confirmed the probable replacement of calcium in the nHAp with the smaller zinc ion owing to substitutional strain in the lattice [44]. The XRD peaks of Sr/Zn-nHAp were also broadened, the broadening was significant in Sr/Zn-4-nHAp, which indicated decreased crystallinity (Figure 1c). The apatite crystallinity significantly declined with higher dopants, as evidenced by the width of the diffraction peaks [45,46]. The XRD analysis indicated that Sr/Zn substitution affected the structure and stability, which reduced the crystallinity of nHAp. In the XRD patterns, the phases of the crystal were generally characterized by peaks, and bioactivity was influenced by increased crystallization [38].

#### 3.1.3. Fourier Transform Infrared Spectroscopy Analysis

**Figure 2:**
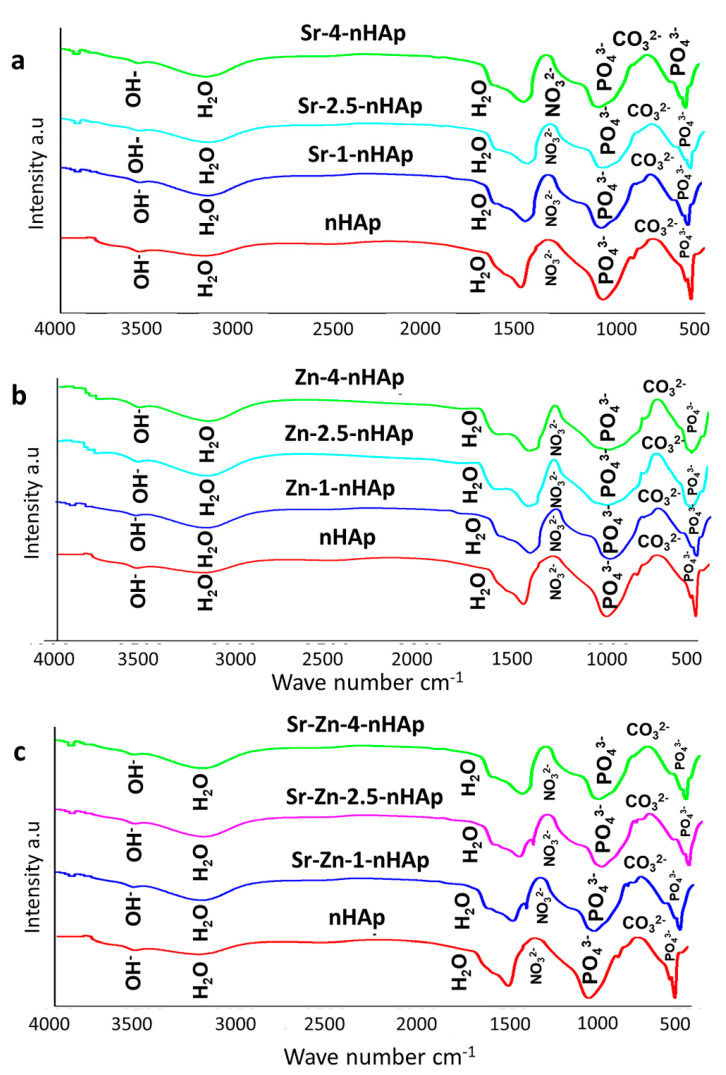
FTIR spectra of Sr-nHAp (**a**), Zn-nHAp (**b**), Sr/Zn-nHAp (**c**).

The FTIR spectra for the n-HAp, Sr-nHAp, Zn-nHAp, and Sr/Zn-nHAp are presented in Figure 2. In the spectra, the obtained bands correspond to phosphate, carbonate, and hydroxyl groups that are distinctive features of hydroxyapatite. The substitution of Sr and Zn ions into the apatite structure was confirmed with the presence of main characteristic bands of hydroxyapatite in all samples, with slight deviations in absorption bands. The majority of the vibrational bands found in the spectra were the characteristic bands for different vibrational modes of the (PO_4_)^3-^ functional group. The bending vibration of the phosphate group was characterized by bands at 562 cm^−1^ [39,47], while the asymmetric stretching mode of (PO_4_)^3^ was affirmed by bands at 1096 cm^−1^ [48]. The presence of carbonate and phosphate groups was confirmed by vibrational bands found in the spectral regions 870–880 cm^−1^ and 1036 cm^−1^ [49]. The incorporation of Sr and Zn ions into the apatite replaced the carbonate group in the pure nHAp, as confirmed by the absence of a band at 889 cm^−1^ in Sr- and Zn-doped nHAp. The bands observed between 1367 cm^−1^ and 1389 cm^−1^ indicated the availability of (NO_3_^-^) groups [50]. The presence of (CO_3_)^2−^ bands at 1450 cm^−1^ and 865 cm^−1^ indicated replacement of the (PO_4_)^3−^ and OH^−^groups in the nHAp lattice by (CO_3_)^2−^ [51]. The presence of adsorbed H_2_O molecules was established by the bands present at 1620 cm^−1^ and 3164 cm^−1^, while peaks between 3523 cm^−1^ and 3621 cm^−1^ represented O-H stretching [52]. Results showed that the FTIR spectra of Sr/Zn-nHAp exhibited a widening of the peaks. A decrease in crystallinity was confirmed by the widening of peaks in metal-doped nHAp composite scaffolds with higher concentrations of strontium and zinc [53]. The incorporation of Sr in the HA lattice was evidenced by a shift to slightly lower wavenumbers as well as peak broadening for the (PO_4_)^3−^ peaks [54]. An increase in both (HPO_4_)^2−^ and (CO_3_) ^2−^ resulted from the substitution of Sr for Ca in the HAp lattice due to the augmented lattice strain initiated by the larger ions of Sr [43].

#### 3.1.4. Transmission Electron Microscopy

**Figure 3:**
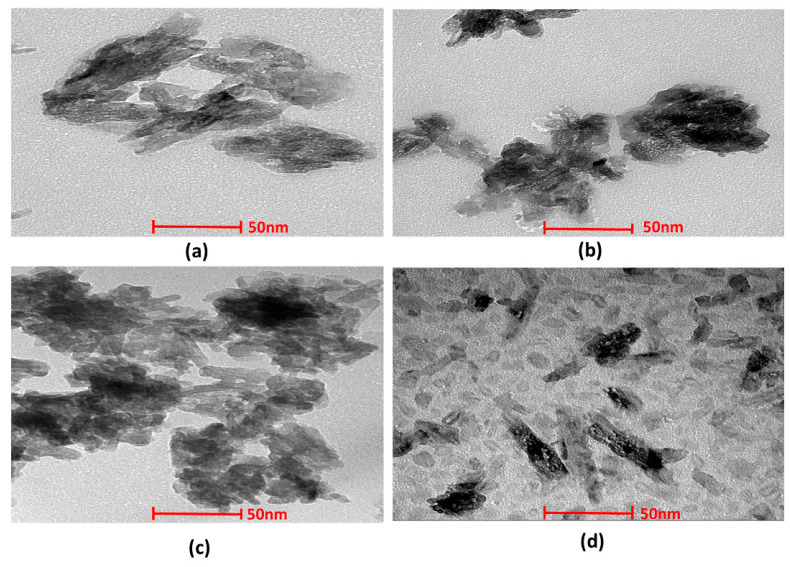
TEM images of nHAp (**a**), Sr/Zn-1-nHAp (**b**), Sr/Zn-2.5-nHAp (**c**), Sr/Zn-4-nHAp (**d**).

**Figure 4:**
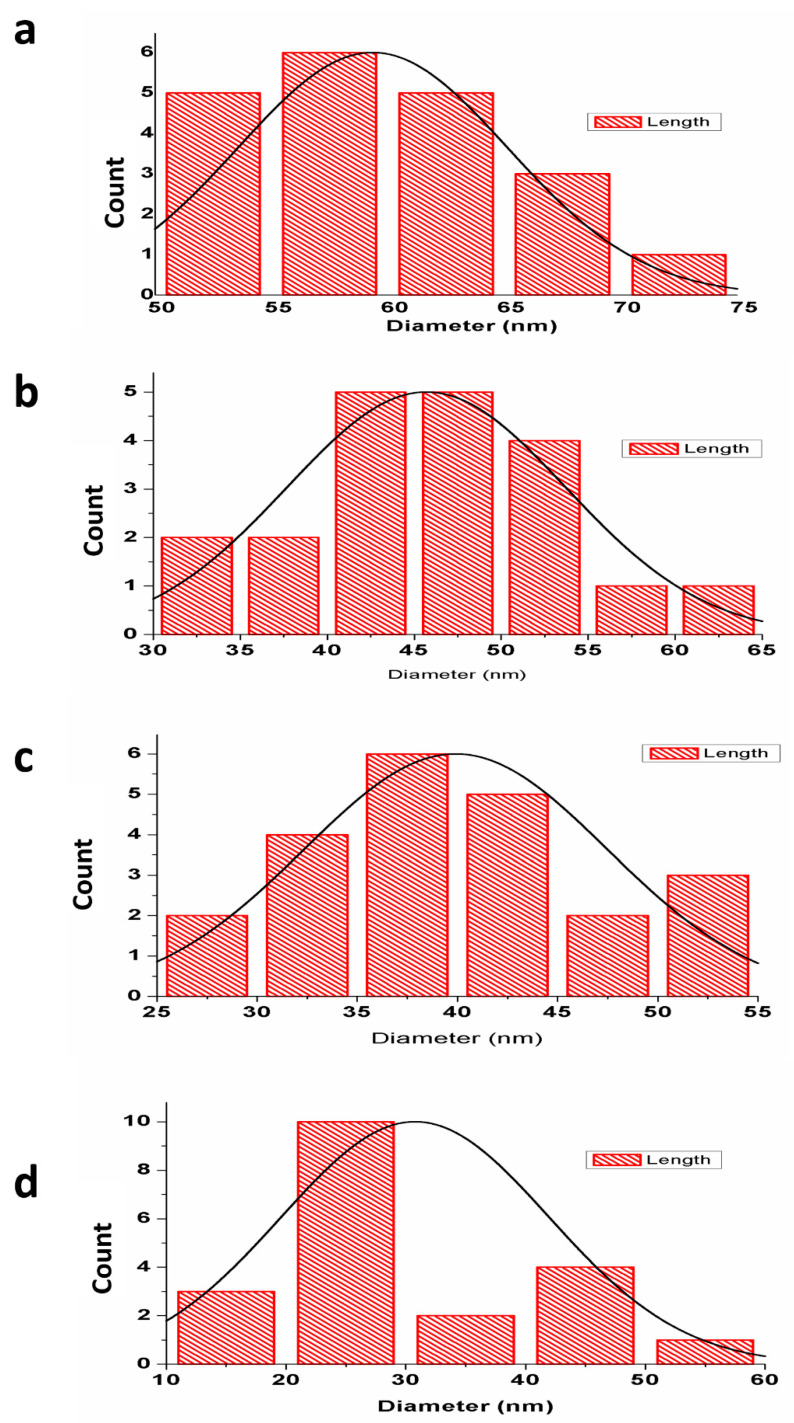
A histogram showing the average particle size obtained from the TEM images of nHAp (**a**), Sr/Zn-1-nHAp (**b**), Sr/Zn-2.5-nHAp (**c**), Sr/Zn-4-nHAp (**d**).

The structural morphology of the nHAp and Sr- and Zn-doped nHAp particles was investigated by TEM. (Figure 3). Morphological analyses showed the presence of aggregated, rod-like hydroxyapatite crystals with a particle size of 50–100 nm. A histogram of the average particle size obtained from the images of TEM is shown in Figure 4. Crystals with equal dimensions were observed in nHAp (Figure 3a). In Sr/Zn-doped nHAp samples along with crystals of equiaxed dimension, rod-like particles are also observed (Figure 3d). The size of the particles was smaller than those of the nHAp, in the range of 50–200 nm. Agglomerated crystal formation with a uniform shape was confirmed by TEM images in Sr/Zn-substituted hydroxyapatite with a size range between 30.77–40.69 nm. The particle size was found to agree with the results of Ferraz et al. (2004) [55,56].

### 3.2. Characterization of Scaffolds after PLGA Incorporation

#### 3.2.1. X-ray Diffraction Analysis of Scaffolds

**Figure 5:**
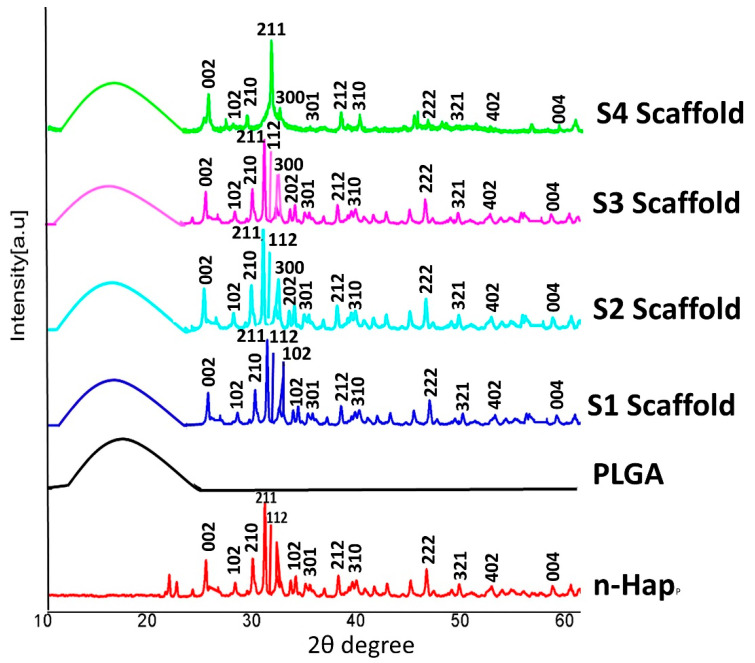
XRD patterns of nHAp, PLGA, and scaffolds. Scaffold 1—PLGA-nHAp, Scaffold 2—PLGA-1% Sr/Zn-nHAp, Scaffold 3—PLGA-2.5% Sr/Zn-nHAp, Scaffold 4—PLGA-4% Sr/Zn-nHAp.

To confirm the crystal phase of the nHAp in the composite scaffolds, XRD characterization was performed (Figure 5). The XRD spectra indicated that the addition of PLGA did not change the composition and crystalline nature of nHAp. The amorphous nature of PLGA copolymer was characterized by the presence of a dome-shaped region at 2θ = 10–25° of the XRD patterns. PLGA incorporation into Sr/Zn-nHAp was confirmed by the presence of a dome-shaped curve in composite scaffolds S1, S2, S3, and S4 (Figure 4). After incorporation of PLGA-Sr/Zn, the composite scaffolds showed the principal diffraction peaks at planes (002), (102), (210), (211), (112), (300), (202), (310), and (222) for the typical crystalline HAp. When the concentration of Sr and Zn were increased, the spectra were not altered, except the intensity, in the scaffolds. Due to the amorphous nature of PLGA, there were no characteristic peaks for the copolymer [57].

#### 3.2.2. FTIR Spectra of Scaffolds

**Figure 6:**
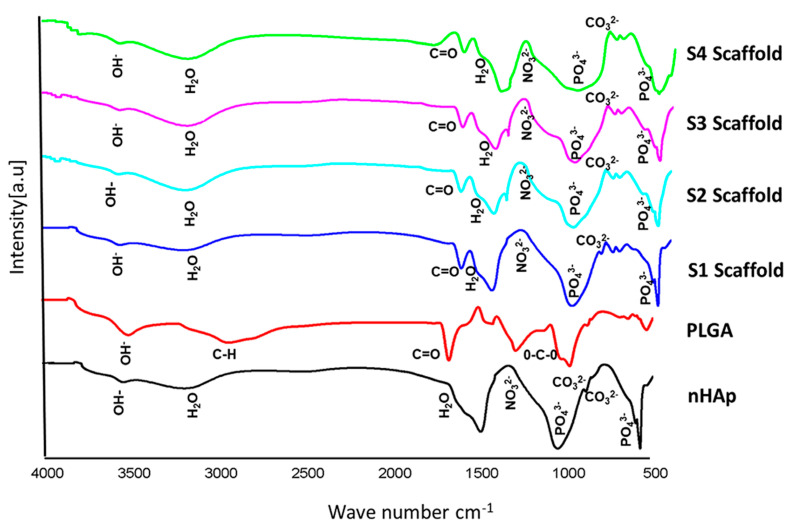
FTIR spectra of nHAp, PLGA, and PLGA-Sr/Zn-nHAp scaffolds. Scaffold 1—PLGA-nHAp, Scaffold 2—PLGA-1% Sr/Zn-nHAp, Scaffold 3—PLGA-2.5% Sr/Zn-nHAp, Scaffold 4—PLGA-4% Sr/Zn-nHAp.

FTIR analysis was performed on the composite scaffolds to ensure cross-linking of PLGA with nHAp and Sr/Zn-nHAp after incorporation with the polymer. FTIR spectra showed the characteristic bands of CO_3_^2−^, PO_4_^3−^, NO_3_^2−^, OH^−^, H_2_O, and –COOH (Figure 5). A 1761 cm^−1^ band was assigned to the C=O, which resulted from the carboxylic acid (COOH) groups in the PLGA [58]. The characteristic bands of nHAp at 569, 866, 1038, 1340, 1624, 3160, and 3570 cm^−1^ were also observed in PLGA-nHAp and PLGA-Sr/Zn-nHAp scaffolds. In the bone scaffold S1 (PLGA-nHAp), the C=O band was detected at 1745 cm^−1^. In the S2 scaffold, (1% Sr/Zn-nHAp incorporated with PLGA), the C=O band was at the level of 1747 cm^−1^. In the FTIR spectra of S3 scaffolds (2.5% of Sr/Zn- nHAp incorporated with PLGA), the C=O band was at the level of 1742 cm^−1^. The C=O band for the S4 scaffolds (4% of Sr/Zn-nHAp incorporated with PLGA) was at the level of 1741 cm^−1^ (Figure 6).

The FTIR pattern of scaffolds supports the results of previous studies [57,59], indicating that hydroxyapatite scaffolds that were incorporated with PLGA showed a slight change in band position in the scaffold, confirming polymer chain incorporation. The change in position of the bands in scaffolds showed that a redshift occurred, demonstrating the formation of hydrogen bonds by cations such as strontium and zinc with C=O groups of PLGA and OH groups of HAp [57,60]. The incorporation of biodegradable polymers with nHAp retains the biocompatibility of the scaffolds and improves the bioresorbability, as required for bone regeneration [61].

#### 3.2.3. TGA Analysis of Scaffolds

**Figure 7:**
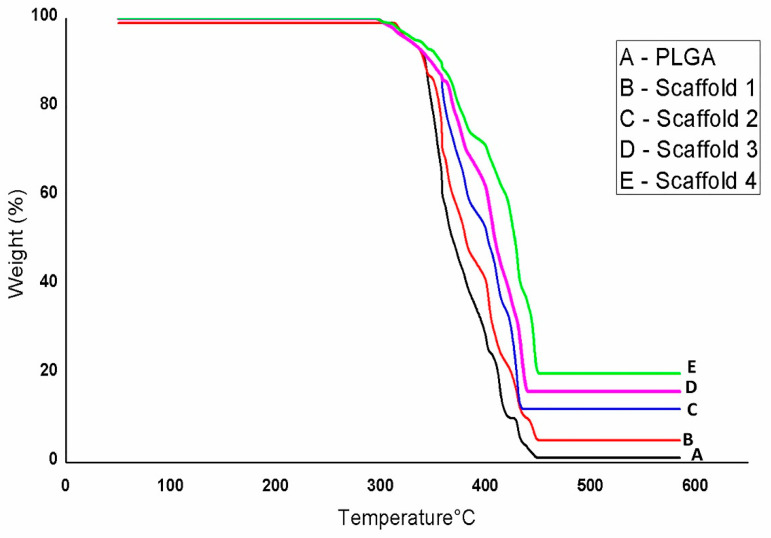
Thermogravimetric analysis (TGA) of nHAp, PLGA, and PLGA-Sr/Zn-nHAp scaffolds. Scaffold 1—PLGA-nHAp, Scaffold 2—PLGA-1% Sr/Zn-nHAp, Scaffold 3—PLGA-2.5% Sr/Zn-nHAp, Scaffold 4—PLGA-4% Sr/Zn-nHAp.

Figure 7 shows the results of thermogravimetric analysis (TGA). The nHAp, PLGA, and composite scaffolds showed different weight losses while increasing temperature. The weight loss of nHAp, PLGA, and scaffolds occurred from 60 °C to 312 °C. The residual weight increased with the addition of nHAp and different concentrations of Sr/Zn to PLGA polymer, which indicated the thermal stability of composite scaffolds at a higher temperature. After 312 ^°^C, nHAp showed a nearly flat curve, and the residual weight was 95%, while PLGA and scaffolds 1, 2, and 3 showed continued weight loss until 450 °C. The weight loss was between 350–600 °C, corresponding to some thermal reactions [62]. Scaffold 4 showed continued weight loss until 470 °C. PLGA showed no residual weight (0%) and the residual weight of scaffold 1 was only 5% after 450 °C. The residual weight of scaffold 2 and scaffold 3 was 15% and 17%, respectively. A higher residual weight (20%) was observed in scaffold 4, which had a higher concentration of strontium and zinc than other scaffolds (Figure 7, Table 4). The mass loss was due to the decomposition of PLGA and other components according to the weight loss observed in TGA curves of composites [63]. The thermal stability of PLGA-nHAp composite scaffolds was confirmed by the elevation of the decomposition temperature from 346 °C to 475 °C following PLGA incorporation to nHAp [64]. 

#### 3.2.4. Scanning Electron Microscopy of Scaffolds

**Figure 8:**
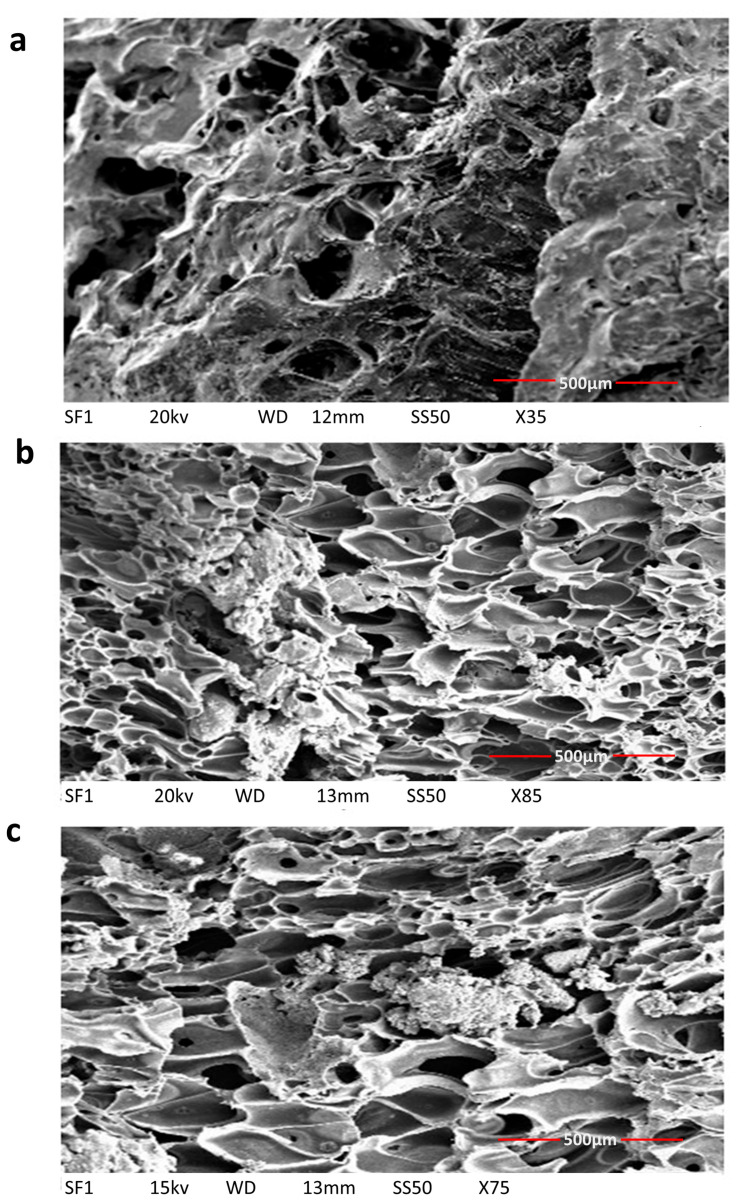
SEM image demonstrating the porous structure of the bone scaffolds. PLGA-nHAp (**a**), PLGA-2.5% Sr/Zn nHAp (**b**), and PLGA-4% Sr/Zn nHAp (**c**).

**Figure 9:**
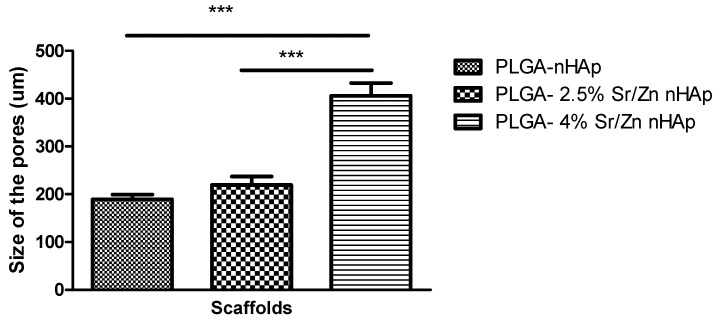
The pore size of the scaffolds (Mean ± SEM). *** *p* < 0.001.

The porous structure of scaffolds was confirmed by the SEM images (Figure 8). The composite scaffolds were interconnected with widely distributed pores. The average pore size for PLGA-nHAp, PLGA-2.5% Sr/Zn-nHAp, and PLGA-4% Sr/Zn-nHAp scaffolds ranged between 189 ± 10.26 to 406 ± 26.54 (Mean ± SEM). The results suggest a statistically significant *p* < 0.0001 increase in the pore size of the composite scaffolds when we doped them with strontium and zinc ions (Figure 9). The pore size should be adequate and interconnected for vascularization and better mass transport of nutrients, gases, and waste [65]. In tissue engineering, the most viable pore sizes for bone cell growth ranked in the range of 150–900 µm [66,67], which enhanced the potential efficacy of the composite materials in the process of bone healing. Though the small pores outside of the reference range are inefficient for the transport of nutrients and can hinder cell invasion and proliferation as well as tissue vascularization, they can greatly enhance the formation of interconnected pores as they can act as channels in between the large pores; meanwhile, larger pores alone may slow down new tissue formation due to reduced surface-to-volume ratios [68].

#### 3.2.5. Bioactivity of Composite Scaffolds

##### PH Measurement

**Figure 10:**
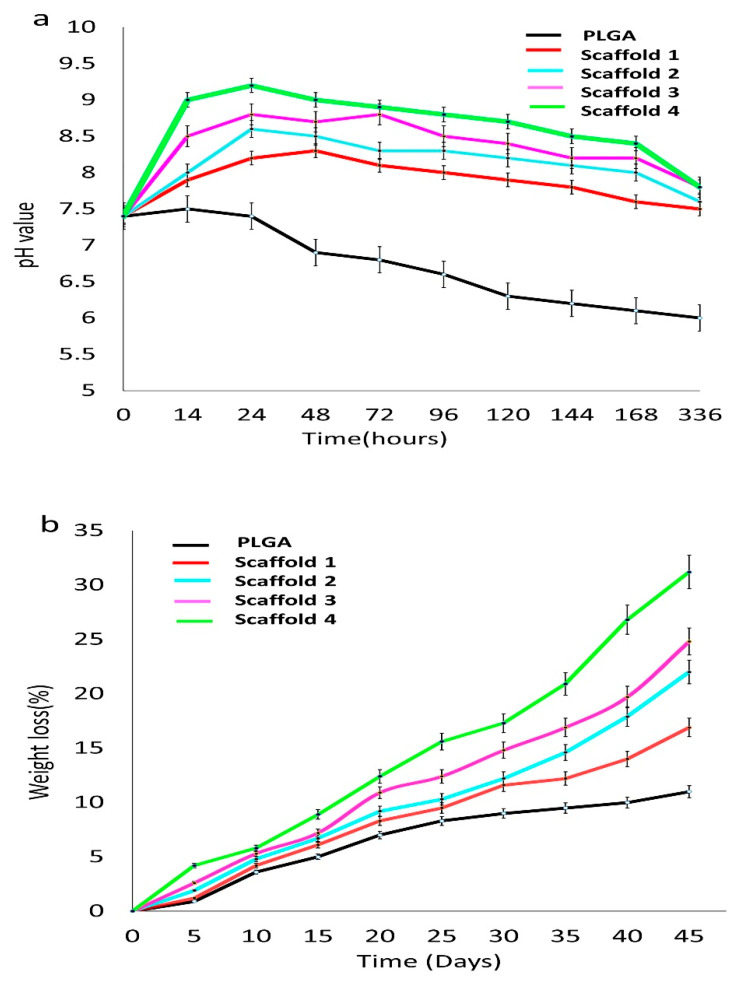
pH value (**a**) and weight loss (**b**) of composite scaffolds immersed in SBF at each time point. Scaffold 1—PLGA-nHAp, Scaffold 2—PLGA-1% Sr/Zn-nHAp, Scaffold 3—PLGA-2.5% Sr/Zn-nHAp, Scaffold 4—PLGA-4% Sr/Zn-nHAp.

The bioactivity of scaffolds was evaluated by measuring pH for PLGA and scaffolds after immersion in SBF. Figure 10 shows the pH values and weight loss of the four scaffolds immersed in SBF at different times. The composite scaffolds initially revealed an elevation in pH followed by a decrease subsequently, attaining an alkaline pH above 7.5. The pH level decline gradually with PLGA degradation within 336 h. In scaffold 1 and scaffold 2, the pH value was increased within 24 h, to 8.2 ± 0.14 (scaffold 1) and 8.6 ± 0.16 (scaffold 2), then decreased slowly to 7.6 ± 0.13 and 7.7 ± 0.09, respectively, at 336 h. At 24 h, the pH values of scaffold 3 and scaffold 4 were highest, at 8.80 ± 0.17 and 9.20 ± 0.19, respectively. Then, the pH value of the two scaffolds decreased to a more alkaline pH of, 7.8 ± 0.20 at 336 h (Figure 10a). The alkaline pH helps in the bone healing process, as it favors mineral deposition [69,70]. The inceptive elevation in the pH of the scaffolds immersed in SBF was a result of the release and exchange of ions from the composites. The bone scaffolds with PLGA and magnesium/strontium were able to maintain the alkaline media, which is necessary for new bone matrix formation [71] as reported in an earlier study [72]. Throughout the degradation period, the pH of SBF for the PLGA-Sr/Zn-nHAp scaffolds was over 7.0, an indication that the composite scaffolds were suitable for bone engineering.

Figure 10b reveals the reduction in weight of PLGA and scaffolds after being immersed in SBF for a different time. It was found that with time, the weight loss of the scaffolds in SBF increased, and the amount of Sr/Zn-nHAp also influenced the weight loss, as scaffold 4 with 4% Sr/Zn-nHAp was most stable. The PLGA polymer showed a decreased weight loss during the entire degradation time. The weight loss of scaffold 1 was slightly faster up to day 21 and then steadied linearly. On day 45, the weight loss of the scaffolds was on the order 16.9%, 22.0%, 24.8%, and 31.2% for scaffolds 1, 2, 3, and 4, respectively. PLGA is reported to degrade by swelling and water absorption, which will lead to ester bond breakage and dissolution of the polymer scaffold fragments [73]. The change in pH of a medium also has a vital role in the weight loss of scaffolds [74]. 

#### Inductive Coupled Plasma-Mass Spectrometry (ICP-MS)

**Figure 11:**
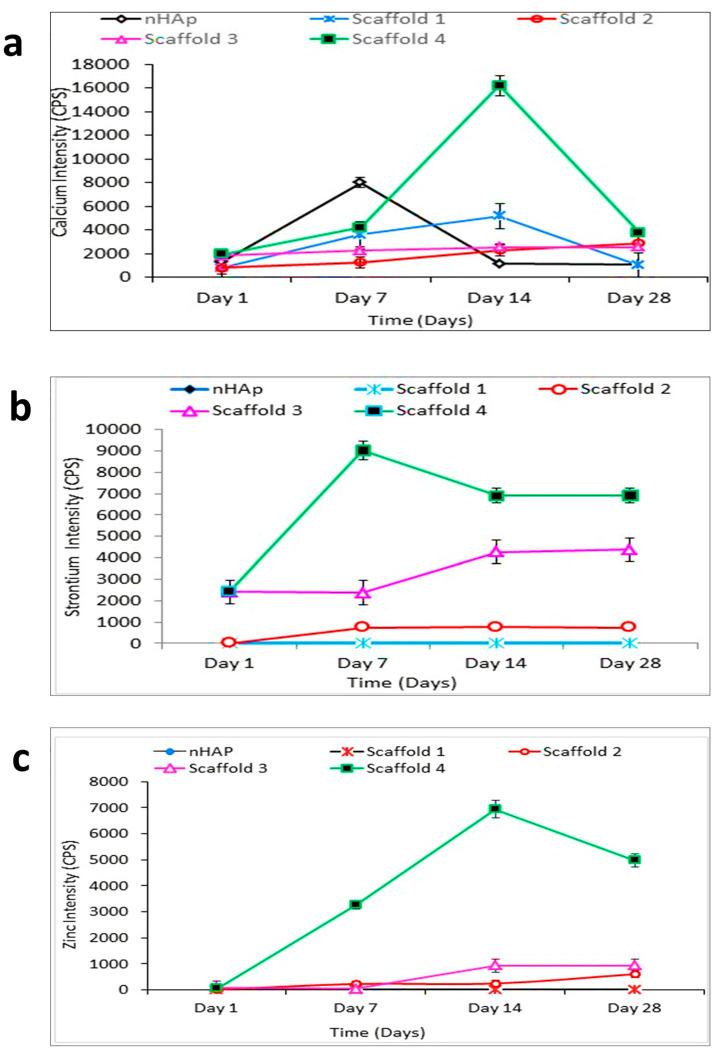
(**a**–**c**) shows the release profile of calcium, strontium and zinc ions from the scaffolds after immersion in SBF. Scaffold 1—PLGA-nHAp, Scaffold 2—PLGA-1% Sr/Zn-nHAp, Scaffold 3—PLGA-2.5% Sr/Zn-nHAp, Scaffold 4—PLGA-4% Sr/Zn-nHAp. The scaffold 4 with higher zinc and strontium concentrations revealed the maximum ion release profiles for calcium (**a**), strontium (**b**) and zinc ions (**c**).

The ion releasing ability of the scaffold after being soaked in the SBF was measured by ICP-MS. Ions were released by the scaffolds, and composite scaffolds with higher zinc and strontium concentrations revealed the maximum ion release profiles. The release profiles of Ca, Zn, and Sr ions are indicated in Figure 11.

Calcium release from the S4 composite scaffolds that contained 4% strontium and zinc were highest, suggesting that the amount of Ca^2+^ release was subordinated to the scaffold composition. The release of Ca^2+^ ions was increased in nHAp samples after 1 week, and the trend of release seemed to decline on day 14 and day 28 (Figure 11a). In the S4 composite scaffold, Ca^2+^ ion release increased on day 7, peaked on day 14, and then decreased. The release of strontium ions at day 1 was highest for S3 and S4 scaffold; the highest release was observed on day 7 in the S4 scaffold, after which it gradually decreased and remained steady even after 3 weeks (Figure 11b). The same trend was noticed for zinc ions, with the S4 composite scaffolds showing the highest release. The Zn^2+^ ion release peaked on day 14, but the level of the released zinc was decreased by the 4th week (Figure 11c). Hence, once these scaffolds are implanted in vivo, the release of strontium and zinc will promote osteoinductivity and antibacterial properties [18,19,75].

#### 3.2.6. Characterization of Scaffolds after Immersion in SBF

##### Fourier Transform Infrared Spectroscopy Analysis

**Figure 12:**
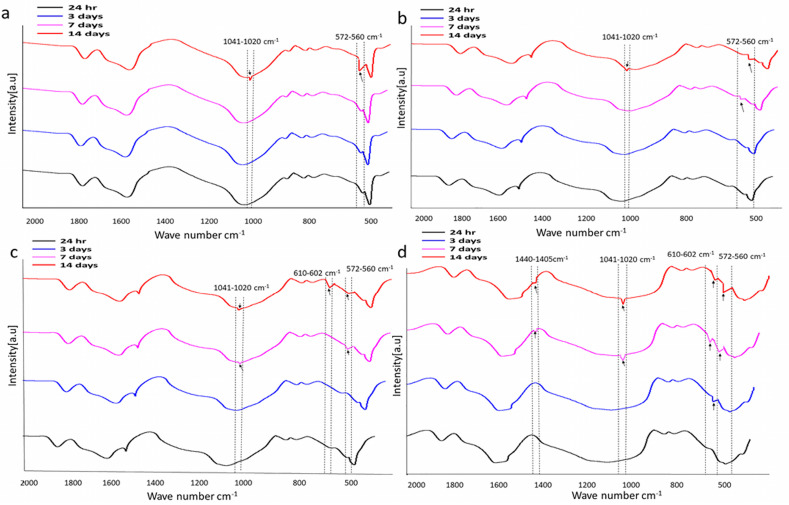
FTIR spectra of composite scaffolds. Scaffold 1—PLGA-nHAp (**a**), Scaffold 2—PLGA-1% Sr/Zn-nHAp (**b**), Scaffold 3—PLGA-2.5% Sr/Zn-nHAp (**c**), Scaffold 4—PLGA-4% Sr/Zn-nHAp (**d**) immersed in SBF for 2 weeks.

Figure 12 shows the FTIR spectra of the scaffolds, which were immersed in SBF for 14 days. After being removed from SBF, FTIR was performed to estimate apatite formation on all scaffolds. No change was noticed in the scaffolds that were removed from SBF after 24 h. The change tended to occur from day 3 and was maintained for up to 14 days in all scaffolds. After 3 days of immersion, the FTIR spectrum exhibited bands at 571 cm^−1^ (O-P-O antisymmetric bending) in scaffold 4 (Figure 12d). Bands at 571cm^−1^ and 1045 cm^−1^ (phosphate group, P-O stretching) were observed in scaffold 3 and 2, respectively, after 7 days (Figure 12c). In scaffold 4, bands at 602 cm^−1^ (O-P-O bending) and 1045 cm^−1^ (P-O stretching) which are characteristic for the phosphate groups, and bands for carbonate groups (1405 cm^−1^, C-O asymmetric stretching) were observed after 7 and 14 days of SBF immersion. In scaffold 1, bands were observed after 14 days. The bands at 560–572 cm^−1^, and 602–610 cm^−1^, and 1020–1041 cm^−1^ are indications of apatite formation [76]. The carbonate bands observed in scaffold spectra indicated that the apatite formed on the surface during immersion in SBF was a carbonated apatite that resembled natural apatite [77]. n-HAp apatite particles present on the surface of polymer/n-HAp membranes act as nucleation sites for mineralization in SBF [78].

##### Scanning and Transmission Electron Microscopy

**Figure 13:**
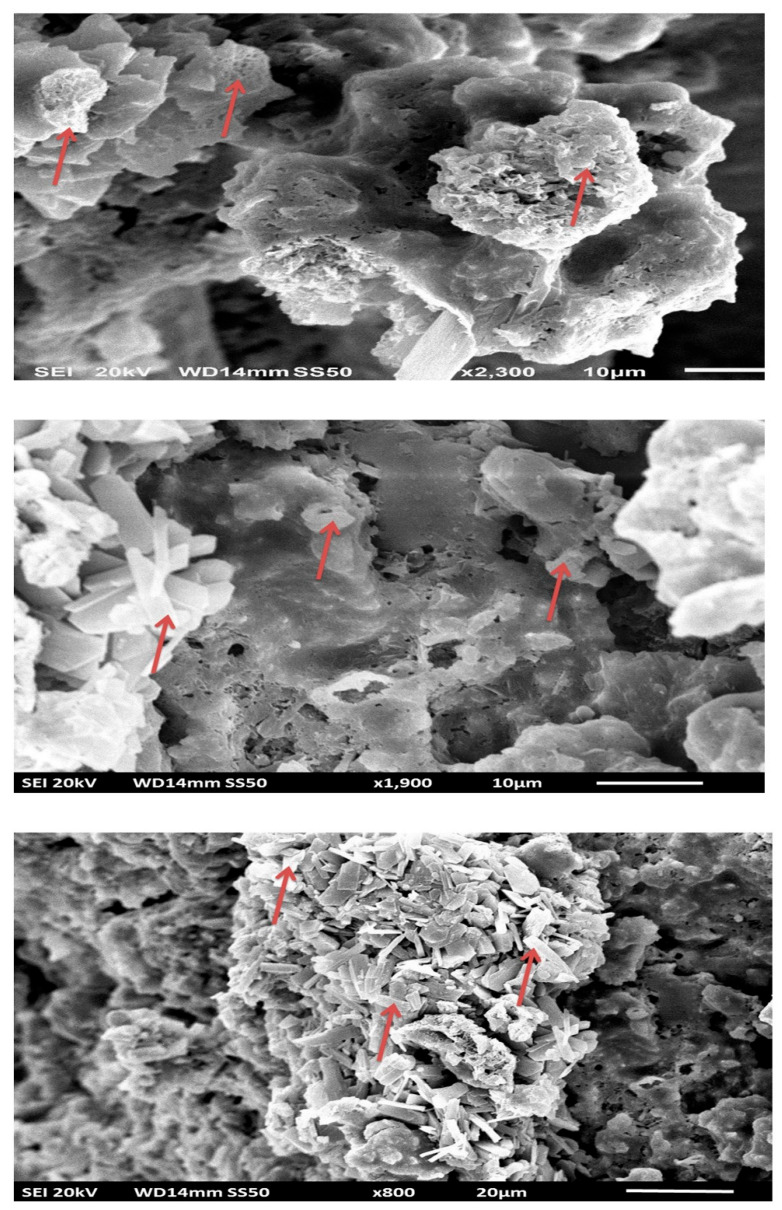
SEM image of crystal formation on the composite scaffold immersed in SBF (crystals shown by arrows).

The bioactivity of composite scaffolds was further investigated by evaluating the formation of apatite on their surfaces in SBF for different time intervals. The morphological changes of scaffolds were observed by SEM and TEM. Composite scaffolds with PLGA and 4% Sr/Zn-nHAp exhibited the most abundant crystal growth after 2 weeks of submersion in SBF (Figure 13). Similar results were reported in an earlier study in which crystals appeared after 2 weeks of scaffold immersion in SBF [79]. Inconsistent with other experimental data, we can conclude that this resultant precipitation was the orthophosphate that formed due to ion release from the bone scaffold.

**Figure 14:**
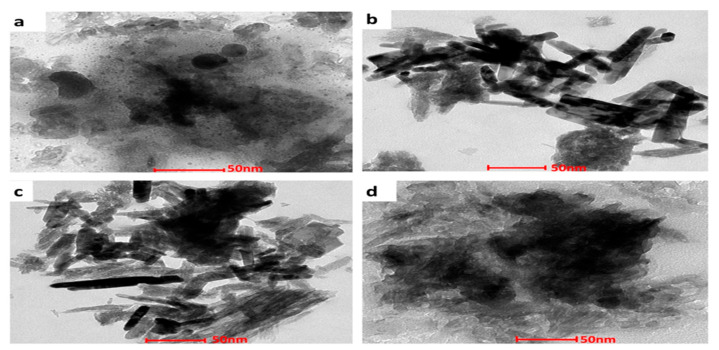
TEM images of Scaffold 1—PLGA-nHAp (**a**), Scaffold 2—PLGA-1% Sr/Zn-nHAp (**b**), Scaffold 3—PLGA-2.5% Sr/Zn-nHAp (**c**), Scaffold 4—PLGA-4% Sr/Zn-nHAp (**d**) submerged in SBF for 2 weeks.

The crystals formed on the scaffolds were examined with TEM, which showed that crystals on scaffolds 1 and 2 were flake-like shapes, but on scaffolds 3 and 4, had denser, spongy shapes (Figure 14). Strontium influenced the biological formation of apatite in SBF at early stages [80]. In Sr-containing HAp ceramics, more apatite layer was formed on the surface with Sr content below 10mol% than on pure HA due to the higher dissolution rate of the composite in SBF [81]. At high doping levels above 10mol%, strontium inhibited the growth of apatite crystals by the replacement of Ca^2+^ with Sr^2+^, which affected the crystallinity, promoted the formation of spherical aggregate on the scaffold, and influenced bone growth [82,83]. Strontium and zinc present in the composite scaffold play an important role in many enzymatic pathways in the human body. Strontium in the form of strontium ranelate is prescribed for its antiresorptive and anabolic properties to osteoporotic patients [84]. The PLGA also enhances the formation of calcium phosphate crystals, a precursor of hydroxyapatite on scaffold surface after submersion in SBF [85].

### 3.3. Mechanical Properties of the Scaffolds

**Figure 15:**
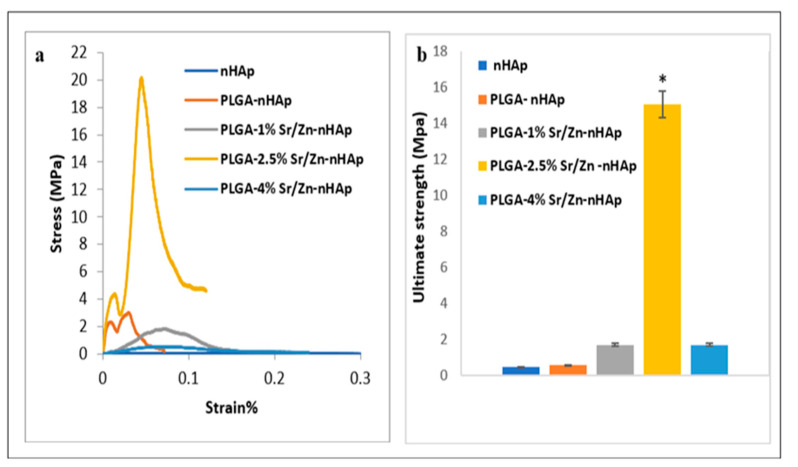
Mechanical testing of different fabricated scaffolds. (**a**) Stress-strain curve, (**b**) ultimate strength. Data is represented as the means ± SEM; n = 3. * = *p* < 0.05.

Mechanical properties of different fabricated scaffolds were assessed using the compressive mechanical test. Results showed different stress-strain curves for the different scaffolds as indicated in Figure 15a. The averages for the ultimate strength of different scaffolds were 0.44 ± 0.32, 0.5 ± 0.94, 1.69 ± 0.5, 15.06 ± 3.05 and 1.68 ± 0.73 MPa for nHAp, PLGA-nHAp, PLGA-1%Zn/Sr-nHAp, PLGA-2.5% Zn/Sr-nHAp and PLGA-4% Zn/Sr-nHAp, respectively (Figure 15b). Data showed that doping nHAp with 2.5% Zn/Sr increased the scaffold ultimate strength by 37%, while further increases in Zn/Sr concentration resulted in a remarkable drop in ultimate strength, indicating that incorporation of Zn/Sr at 2.5% enhanced the scaffold mechanical properties while further increases to 4% had a negative effect on the scaffold mechanical properties. The drastic drop in 4% Zn/Sr-doped nHAp ultimate strength could be a result of poor dispersion between PLGA and Zn/Sr nHAp, which was also indicated by the brittle fracture observed in the surface of the scaffold during the test.

Previous studies reported a compressive strength of HAp-PLGA of 2.8 MPa, which was closely similar to our results [86]. Liu et al. [87] reported that the poor mechanical properties of PLGA and HAp composites resulted from difficulties in dispersion, while a better-dispersed scaffold of polymer and bioceramic resulted in higher mechanical strength compared to the non-well dispersed scaffold. Another study also showed that incorporation of zinc and metal ions into HAp at higher concentrations increased the amount of substitution of Ca by Zn and changed the Ca/P ratio, potentially resulting in Ca-deficient HAp and changes in structure, which was also in accordance with our XRD results [76].

## 4. Limitation of the Study

We would like to report certain limitations of this study as well. The drastic decrease in the mechanical properties after increasing Zn/Sr concentrations to 4% was unexpected, and it could be a result of the reduced crystallinity and shrinkage of the nHAp due to a larger proportion of Zn/Sr substituting Ca/P or due to the bad dispersion and agglomeration of these ions; therefore, adapting better techniques for scaffold homogenization and mixing are required. The scaffolds were examined for porosity in 2D only using TEM and SEM; the results could be supplemented by a micro-CT study. Only strontium and zinc were added to the scaffolds to augment bone formation; in future study, metal oxide nanoparticles such as Fe_3_O_4_ could also be added to create a nanoscale magnetic field that can activate numerous cell-signaling pathways and promote osseointegration. TGA analysis was carried out to check the thermal stability of the composite scaffolds, and it was shown that composite scaffolds with the highest amounts of strontium and zinc were most stable. However more detailed TGA analysis would be helpful to compute the first and second derivatives, which will identify if primary and secondary mass losses are occurring. The presence of several mass losses can be attributed to the degradation of the nanoparticles. Further study on degradation kinetics would explain the variation of the degradation rate against Sr and Zn content.

## 5. Conclusions

Bioactive composite scaffolds with different concentration of Sr/Zn-nHAp and PLGA (as the matrix) were developed. The XRD pattern and FTIR spectra revealed the phase composition and crystal properties of nHAp in both Sr/Zn-doped powders and composite scaffolds and also confirmed the incorporation of PLGA in scaffolds. ICP-MS study confirmed the release of strontium and zinc ions, which are known to be anabolic to bone, and additionally, the zinc-containing composite scaffolds known to have antibacterial activity. Crystallinity decreased while increasing the Sr/Zn concentration, and composite scaffolds with less crystalline nHAp produced a bioactive layer that was suitable for bone regeneration. The scaffolds were able to form an orthophosphate layer on the surface when immersed in simulated body fluid; this was supported by SEM and TEM results. Moreover, in vitro degradation studies demonstrated that Sr/Zn-nHAp-incorporated PLGA degradation led to increases in the pH of SBF and accelerated the weight loss rate of PLGA. Scaffold 4, with the high concentration of Sr/Zn, was less crystalline, thermostable, biodegradable, and had more orthophosphates on the scaffold surface. PLGA-2.5% Zn/Sr-nHAp (scaffold 3) showed the best ultimate strength, which was closely similar to that of cancellous bone.

The current scaffolds fulfill the criteria defined for an ideal scaffold, as they are bioactive, biodegradable, porous, and mechanically stable. In vivo implantation of these scaffolds in small laboratory animals would be a further step towards translational medicine.

Future work will be directed towards using immunomodulating strategies such as the inclusion of anti-inflammatory drugs and growth factors in these scaffolds, which would promote early and successful bone regeneration.

## Figures and Tables

**Table 1:** The composition of the Sr- and Zn- doped nHAp powder (mol %).

Sample	Sr (mol %)	Zn (mol %)	nHAp (mol %)
Sr-1-nHAp	1.0	0.0	1.0
Sr-2.5-nHAp	2.5	0.0	1.0
Sr-4-nHAp	4.0	0.0	1.0
Zn-1-nHAp	0.0	1.0	1.0
Zn-2.5-nHAp	0.0	2.5	1.0
Zn-4-nHAp	0.0	4.0	1.0
Sr/Zn-1-nHAp	1.0	1.0	1.0
Sr/Zn-2.5-nHAp	2.5	2.5	1.0
Sr/Zn-4-nHAp	4.0	4.0	1.0

**Table 2:** Composition of PLGA-incorporated Sr-Z-n-HAp bone scaffolds.

Bone Scaffolds	PLGA (mol %)	Sr (mol %)	Zn (mol %)	nHAp (mol %)
Scaffold 1 (S1)	3.0	0.0	0.0	1.0
Scaffold 2 (S2)	3.0	1.0	1.0	1.0
Scaffold 3 (S3)	3.0	2.5	2.5	1.0
Scaffold 4 (S4)	3.0	4.0	4.0	1.0

**Table 3:** Particle size analysis of nHAp, Sr-nHAp, Zn-nHAp, Sr/Zn-nHAp.

Samples	Particle Size (nm)		
	d_10_	d_50_	d_90_
nHAp	58.6	92.3	196.4
Sr-1-nHAp	52.2	86.0	190.3
Zn-1-nHAp	48.4	89.0	185.4
Sr-2.5-nHAp	43.7	86.0	167.6
Zn-2.5-nHAp	44.5	86.0	163.0
Sr-4-nHAp	40.3	84.4	152.6
Zn-4-nHAp	42.3	85.0	150.8
Sr/Zn-1-nHAp	45.2	79.4	146.8
Sr/Zn-2.5-nHAp	47.5	80.2	148.2
Sr/Zn-4-nHAp	46.4	76.6	149.0

**Table 4:** TGA weight loss temperatures and residues of the scaffolds.

Sample	T2% (°C)	T 10% (°C)	T 50% (°C)	T 90% (°C)	Residue (wt%)
nHAp	532		-	-	95
PLGA	333	338	369	425	0
PLGA nHAp	345	344	380	432	5
PLGA nHAp 1% Zn Sr	350	360	402	522	15
PLGA nHAp 2.5% Zn Sr	358	365	410	555	17
PLGA nHAp 4% Zn Sr	369	372	421	572	20

Scaffold 1—PLGA-nHAp, Scaffold 2—PLGA-1% Sr/Zn-nHAp, Scaffold 3—PLGA-2.5% Sr/Zn-nHAp, Scaffold 4—PLGA-4% Sr/Zn-nHAp.

## Data Availability

All the relevant data is provided in the paper. Links to the Supplementary Figures can be found in Supplementary Figures S1 and S2.

## Supplementary Materials

The following supporting information can be downloaded at: https://www.mdpi.com/article/10.3390/jfb13010013/s1, Figure S1: showing apparatus used for the ScCO_2_ system. (a) ISCO SFX-220 extraction system red arrows shows the holder with a mold containing a sample. (b) Holder with a mold for placing a sample. (c) Mold use for making scaffolds. (d) Scaffolds prepared for compression testing; Figure S2: Particle size bar diagram.
