# Supplementary material for: Biomimetic PLGA/Strontium-Zinc Nano Hydroxyapatite Composite Scaffolds for Bone Regeneration
Source: J Funct Biomater. 2022 Jan 28;13(1):13. doi: 10.3390/jfb13010013 (PMC8883951; doi:10.3390/jfb13010013)
Supplement: Supplementary file 1 [file jfb-13-00013-s001.zip › jfb-1529647-supplementary.pdf]

Journal of
Functional
Biomaterials

MDPI

Supplementary

Biomimetic PLGA/Strontium-Zinc Nano Hydroxyapatite Composite Scaffolds for Bone Regeneration

Mozan Hassan [1,†], Mohsin Sulaiman [1,†], Priya Dharshini Yuvaraju [2], Emmanuel Galiwango [3,4], Ihtesham ur Rehman [5], Ali H. Al-Marzouqi [3], Abbas Khaleel [6] and Sahar Mohsin [1,*]

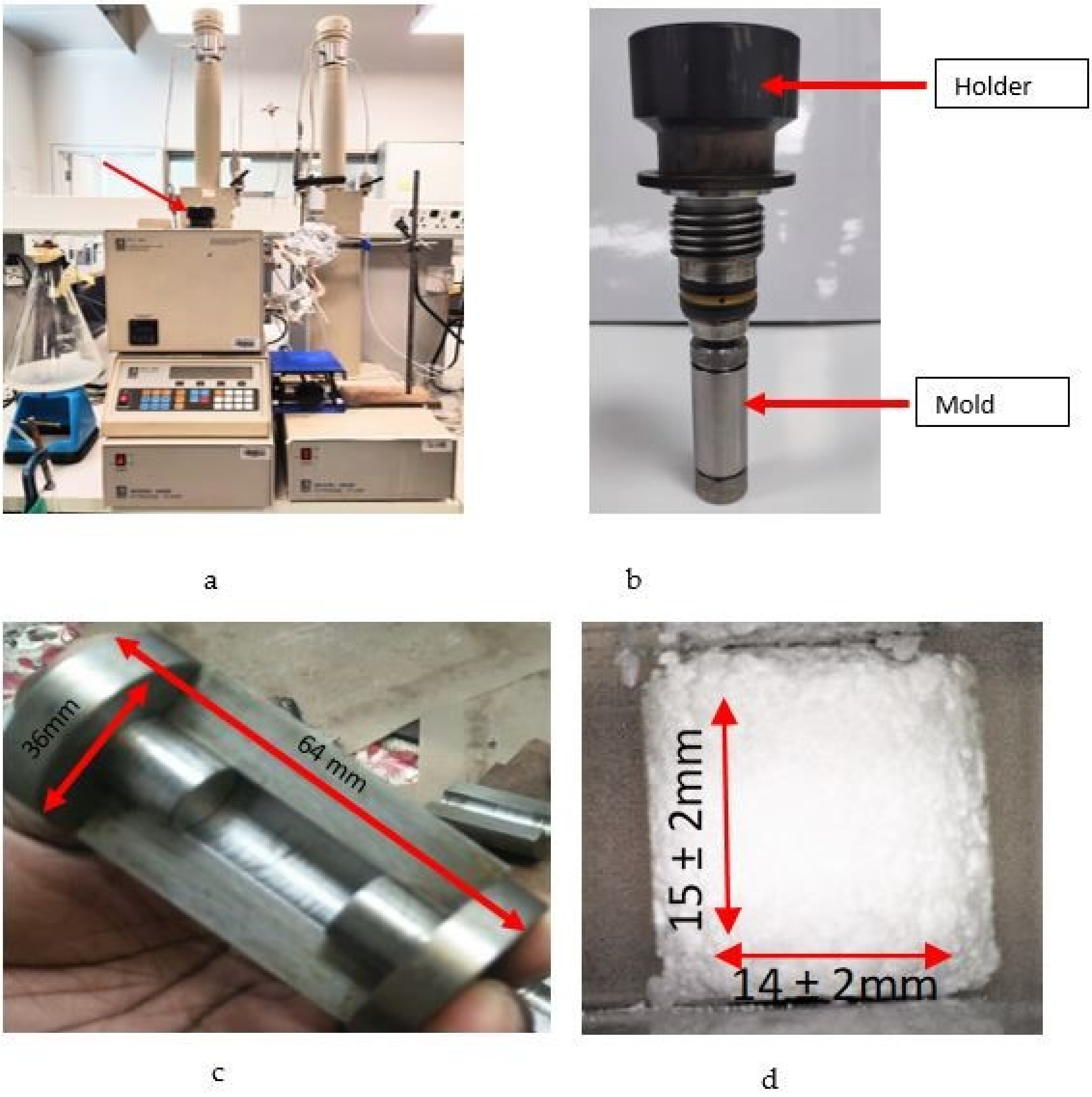

Figure S1. Showing apparatus used for the ScCO2 system. (a) ISCO SFX-220 extraction system red arrows shows the holder with a mold containing a sample. (b) Holder with a mold for placing a sample. (c) Mold use for making scaffolds. (d) Scaffolds prepared for compression testing.

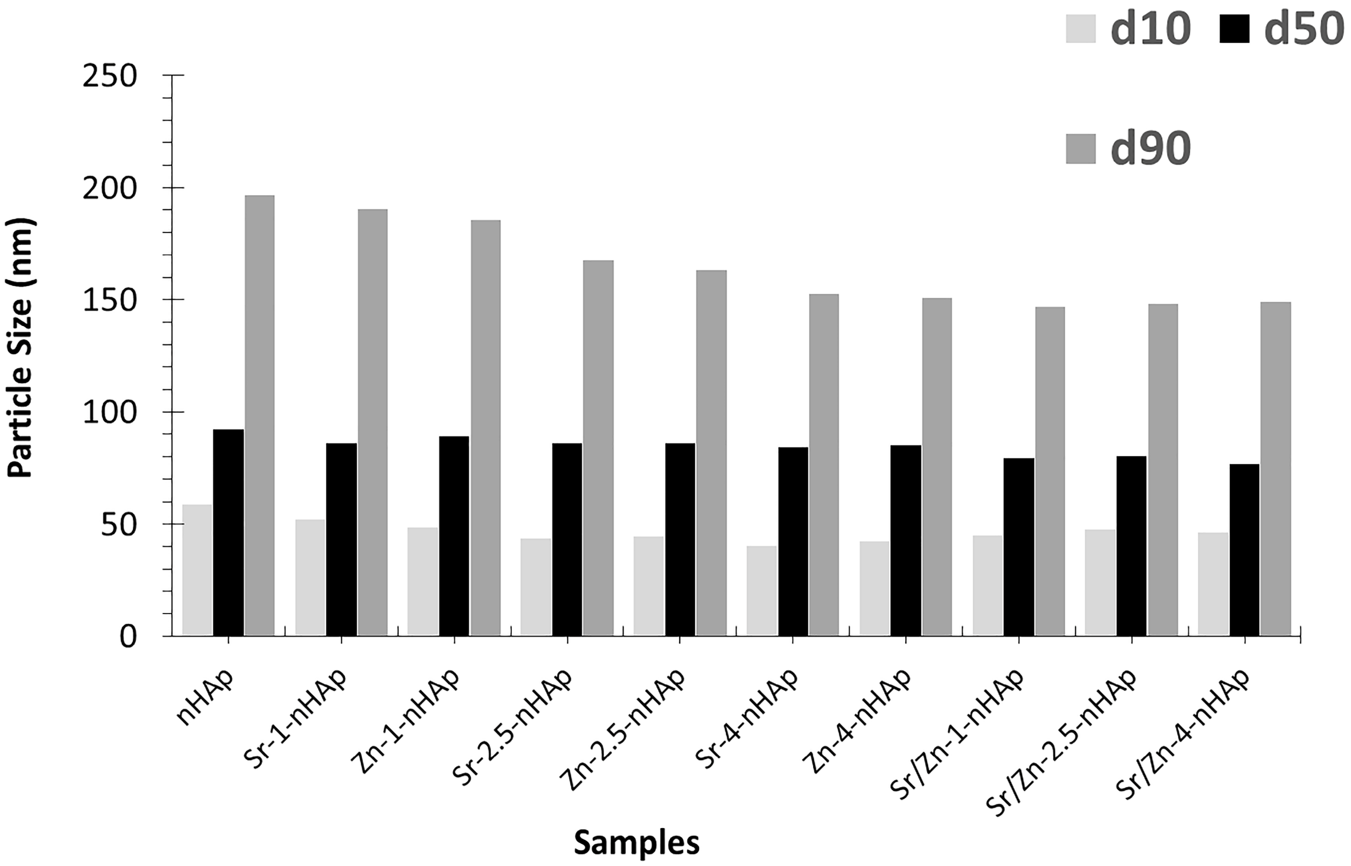

Figure S2. Particle size bar diagram.
